# Preparation and Piezocatalytic Performance of γ-AlON Particles for Dye-Pollutant Degradation Under Ultrasonic Vibration

**DOI:** 10.3390/molecules29235698

**Published:** 2024-12-02

**Authors:** Dan Zhu, Yanyan Wang, Le Xiao, Yu Dai, Jian Wu

**Affiliations:** 1 International Institute for Materials Innovation, Nanchang University, Nanchang 330031, China; a4236623@126.com (D.Z.); yanyanwang7@126.com (Y.W.); lexiao@email.ncu.edu.cn (L.X.); 2 School of Physics and Materials Science, Nanchang University, Nanchang 330031, China; 3 Advanced Corporation for Materials and Equipments Co., Ltd., ACME, Changsha 410118, China

**Keywords:** γ-AlON particles, piezocatalysis, dye pollutants, ultrasonic vibration

## Abstract

Piezocatalytic materials have attracted widespread attention in the fields of clean energy and water treatment because of their ability to convert mechanical energy directly into chemical energy. In this study, γ-AlON particles synthesised using carbothermal reduction and nitridation (CRN) were used for the first time as a novel piezocatalytic material to degrade dye solutions under ultrasonic vibration. The γ-AlON particles exhibited good performance as a piezocatalytic material for the degradation of organic pollutants. After 120 min under ultrasonic vibration, 40 mg portions of γ-AlON particles in 50 mL dye solutions (10 mg/L) achieved 78.06%, 67.74%, 74.29% and 64.62% decomposition rates for rhodamine B (RhB), methyl orange (MO), methylene blue (MB) and crystal violet (CV) solutions, respectively; the fitted k values were 13.35 × 10^−3^, 10.79 × 10^−3^, 12.09 × 10^−3^ and 8.00 × 10^−3^ min^−1^, respectively. The piezocatalytic mechanism of γ-AlON particles in the selective degradation of MO was further analysed in free-radical scavenging activity experiments. Hydroxyl radicals (•OH), superoxide radicals (•O_2_^−^), holes (h^+^) and electrons (e^−^) were found to be the main active substances in the degradation process. Therefore, γ-AlON particles are an efficient and promising piezocatalytic material for the treatment of dye pollutants.

## 1. Introduction

Owing to the rapid pace of global industrialisation, the problem of environmental pollution has escalated significantly worldwide. Organic pollutants are progressively permeating natural environments such as water, air and soil, exerting adverse effects on ecosystems and ultimately affecting human health [1,2]. Hence, effective methods to degrade organic pollutants are urgently needed.

In recent years, piezocatalytic materials have attracted widespread attention in the fields of clean energy and water treatment because they can convert mechanical energy, such as vibration, friction and the movements of natural winds and tides, directly into chemical energy [3]. This approach harnesses external mechanical energy to activate piezoelectric materials, generating a piezoelectric potential and inducing charges to produce reactive free radicals, thereby facilitating the degradation of organic pollutants [4,5]. Numerous overlooked and underutilised natural sources of mechanical energy can be harnessed for this purpose [6,7], positioning piezoelectric catalytic technology as a sustainable and energy-efficient catalytic solution. Piezocatalytic materials can be categorised into groups of traditional materials, such as perovskites [8,9,10], nonperovskite zincblendes [11,12], piezoelectric polymers [13,14], and novel piezoelectric materials [15,16,17,18,19]. These catalysts enable the degradation of organic pollutants through mechanical energy-induced electrochemical oxidation–reduction reactions without reliance on light or electricity [20]. However, the synthesis methods for most piezocatalytic materials are low-yielding and expensive [20,21,22].

γ-AlON, a solid solution within the Al_2_O_3_-AlN binary system, is well-known for its exceptional physicochemical properties [23,24]. A stable γ-AlON is a non-stoichiometry compound and defect-rich structure. Both theoretical calculations and experimental results reveal that aluminium vacancies occupy the octahedral sites and play a predominant role [25,26,27]. It is widely recognized that defect engineering is an effective method to enhance piezocatalytic performance [28,29,30]. The synthesis methods of γ-AlON powder include the solid-state reaction method (SSR) [31,32] and carbothermal nitridation method (CRN) [33,34]. The CRN method has the advantages of low-cost raw materials, simple process and high output [34,35]. The characteristics indicate that γ-AlON is a potential low-cost piezocatalytic material. However, to the best of our knowledge, there is no research on the piezocatalytic performance of γ-AlON.

In this study, γ-AlON was prepared using carbothermal reduction and nitridation (CRN). The effects of the catalyst dose and dye type (rhodamine B (RhB), methyl orange (MO), methylene blue (MB) and crystal violet (CV)) on the catalytic degradation efficiency and rate constant k under the ultrasonic vibration excitation piezoelectric effect were systematically studied. The piezocatalytic mechanism of the γ-AlON particles was investigated by analysing the generation of active substances such as hydroxyl radicals (•OH), superoxide radicals (•O_2_^−^), holes (h^+^) and electrons (e^−^) in free-radical scavenging-activity experiments.

## 2. Results and Discussion

### 2.1. Properties of γ-AlON Particles

**Figure 1:**
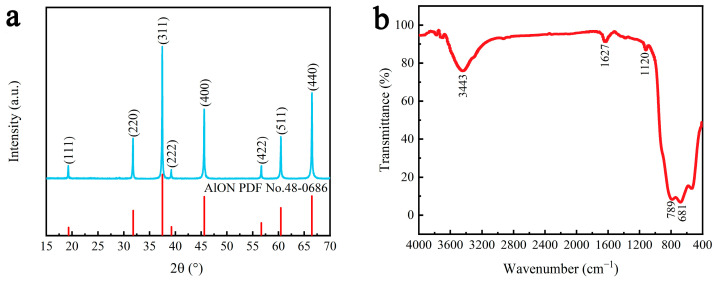
(**a**) XRD and (**b**) FTIR patterns of γ-AlON particles.

The observed diffraction peaks at 19.3° (111), 31.8° (220), 37.5° (311), 39.2° (222), 45.6° (400), 56.7° (422), 60.5° (511) and 66.5° (440) were consistent with the face-centred-cubic spinel structure of γ-AlON (PDF#48-0686) (Figure 1a). Figure 1b shows the FTIR pattern of the samples. The absorption peaks at 681 and 789 cm^−1^ correspond to the stretching vibrations of the Al–N and Al–O groups, respectively [36], which confirms the synthesis of AlON. The absorption peak at 1627 cm^−1^ corresponds to the adsorbed water on the sample surface [37], while the absorption peak at 3443 cm^−1^ corresponds to the stretching vibration of the O–H groups from the adsorbed water [38]. The absorption peak at 1120 cm^−1^ corresponds to the stretching vibration of the C–O groups [39], which may be attributed to the dissolution of CO_2_ from air.

**Figure 2:**
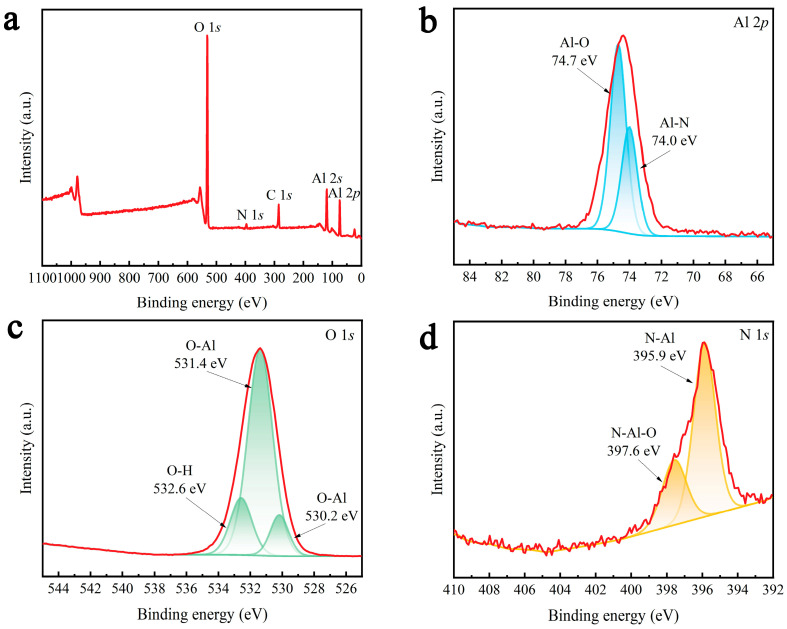
(**a**) XPS spectra of γ-AlON particles and enlarged signals for (**b**) Al 2*p*, (**c**) O 1*s*, (**d**) N 1*s*.

Figure 2a shows the XPS spectra of γ-AlON particles. The Al 2*p* peaks at binding energies of 74.0 and 74.7 eV correspond to the characteristic peaks of Al–N and Al–O, respectively (Figure 2b) [40]. The O 1*s* peaks at binding energies of 530.2 and 531.4 eV were characteristic of O–Al, and 532.6 eV corresponds to the characteristic peak of O–H (Figure 2c) [41], which may be attributable to the O–H groups on the sample surface or those from adsorbed water. The N 1*s* binding energies of 395.9 and 397.6 eV correspond to the characteristic peaks of N–Al and N–Al–O, respectively (Figure 2d) [42].

**Figure 3:**
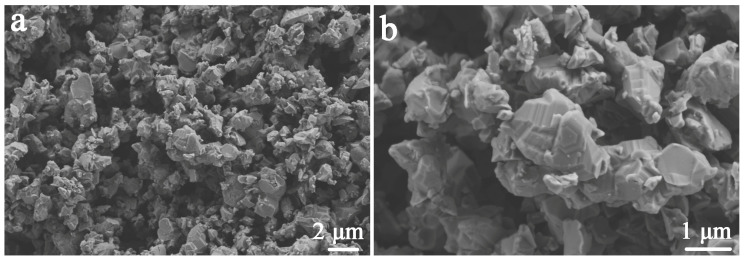
SEM images of γ-AlON particles. (**a**) morphology; (**b**) morphology of surface step.

Figure 3a,b shows the morphology of the γ-AlON particles, revealing good dispersion with no agglomeration of the irregular grains. The rich step surfaces of the γ-AlON particles observed in Figure 3b are typical morphological features of γ-AlON.

**Figure 4:**
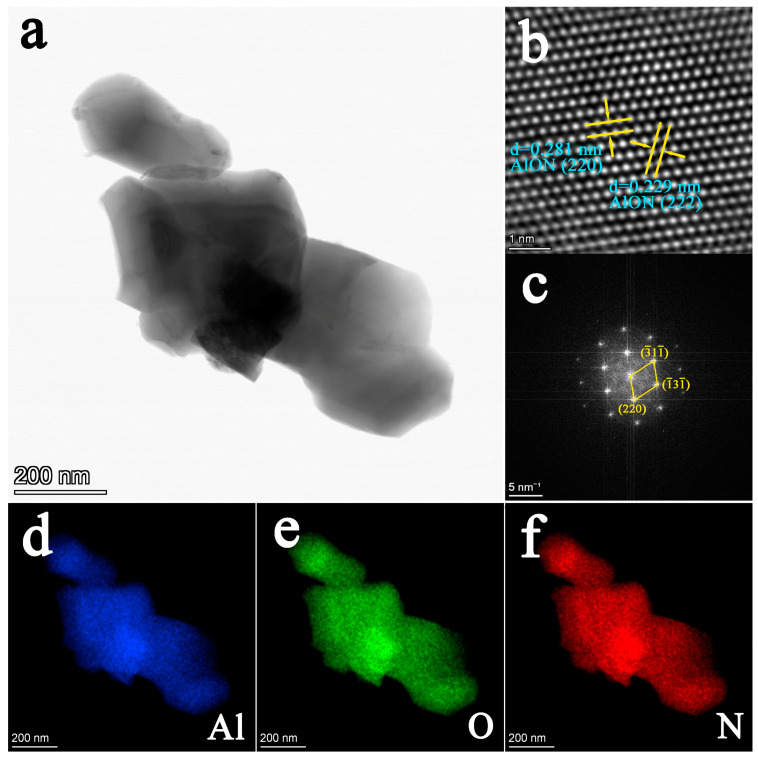
(**a**) TEM, (**b**) HRTEM and (**c**) SAED and element mapping for (**d**) Al, (**e**) O and (**f**) N of γ-AlON particles.

TEM and HRTEM were employed to further observe the γ-AlON particles. The particle morphology observed in Figure 4a was consistent with the scanning electron microscopy image of the γ-AlON particles. The spacing of the two lattice planes indicated in Figure 4b was estimated to be 0.281 and 0.229 nm, which is consistent with the *d*-spacing of the (220) and (222) planes of the γ-AlON particles, respectively. The selected area electron diffraction (SAED) pattern was indexed as a face-centred-cubic structure with space group *Fd*$\bar{3}$*m* (Figure 4c), which is consistent with the XRD results (Figure 1a). Moreover, the SAED pattern exhibited clear and sharp diffraction spots, indicating the satisfactory crystallinity of the γ-AlON particles. Figure 4d–f show the element mappings for Al, O and N, indicating uniform element distributions within the γ-AlON particles.

**Figure 5:**
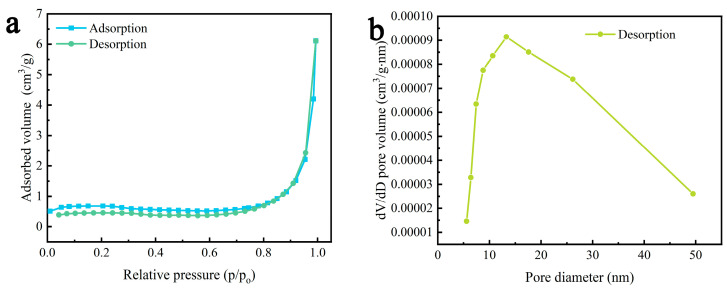
(**a**) Nitrogen adsorption–desorption isotherm and (**b**) pore-size distribution curve of γ-AlON particles.

The adsorption capacity of the catalyst surface is an important factor affecting the evaluation of piezocatalytic performance. The larger the specific surface area, the more obvious the adsorption effect. Figure 5 shows the nitrogen adsorption–desorption isotherm and pore-size distribution curve for the γ-AlON particles. Figure 5a shows a type III isotherm, with a lower adsorption volume at lower relative pressure and a higher adsorption volume at higher relative pressure, which implies the presence of pores in the samples. The pore-size distribution curve (Figure 5b) indicates a mesoporous sample with few pores. The specific surface area of the γ-AlON particles was only 2.73 m^2^/g, indicating that the specific surface area is not a major factor in the catalytic degradation of dye pollutants in this study.

**Figure 6:**
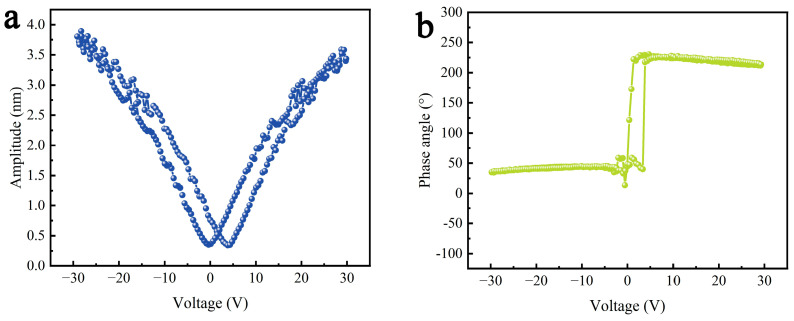
(**a**) Amplitude butterfly curve and (**b**) phase hysteresis loop of γ-AlON particles.

Figure 6 shows the piezoelectric responses of the γ-AlON particles in a periodic triangle wave ranging from −30 to +30 V. Figure 6a,b depict the amplitude butterfly curve and phase hysteresis loop of the γ-AlON particles during the “on” state, which resemble the typical amplitude-voltage and phase-voltage hysteresis loop, respectively. The phase contrast of the γ-AlON particles is approximately 180°, which is a signature of domain reversal in typical ferroelectrics [43]. The typical amplitude-voltage and phase-voltage hysteresis loops are the main factors used to evaluate piezoelectric performance [44,45,46].

### 2.2. Piezocatalytic Degradation of Dye Pollutants

**Figure 7:**
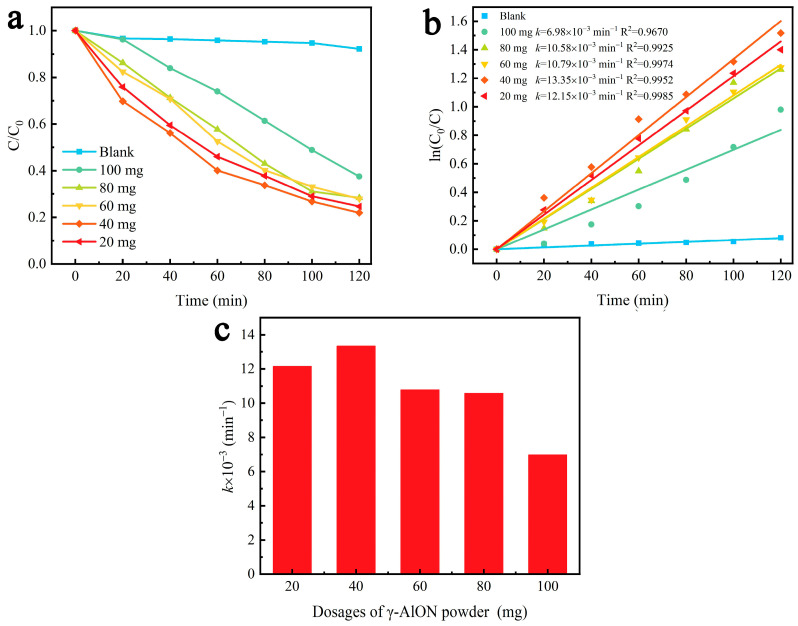
(**a**) The relative concentration curves, (**b**) the first-order reaction kinetics curves and (**c**) the rate constant *k* of RhB solutions with different dosages of γ-AlON particles under ultrasonic vibration.

Figure 7a shows the relative concentration (*C*/*C*_0_) of RhB solutions under ultrasonic vibration in the presence of γ-AlON particles. RhB solution without γ-AlON particles exhibited a degradation rate of only 7.79% after 120 min under ultrasonic vibration. When γ-AlON particles were added, the concentration of the RhB solution gradually decreased with increasing vibration time. In the presence of 20, 40, 60, 80 and 100 mg of γ-AlON particles, the RhB solutions exhibited 75.35%, 78.06%, 72.11%, 71.67% and 62.48% degradation rates after 120 min under ultrasonic vibration, respectively. These results demonstrated that the decomposition efficiency of RhB solutions initially increased and subsequently decreased with increasing doses of γ-AlON particles, indicating that the presence of excessive catalyst decreased the piezocatalytic effect. Figure 7b shows the linear plot of ln (*C*_0_/*C*) versus time for the piezocatalytic effect. The fitting curves indicate a linear relationship between percent degradation and vibration time, indicating that the degradation process obeys first-order kinetics. The slope *k* of the fitting curves can be used to characterise the reaction rate [47]. The *k* values were 12.15 × 10^−3^, 13.35 × 10^−3^, 10.79 × 10^−3^, 10.58 × 10^−3^ and 6.98 × 10^−3^ min^−1^ for γ-AlON particle doses of 20, 40, 60, 80 and 100 mg, respectively (Figure 7c). These results indicate that excess γ-AlON particles can reduce the rate of piezocatalytic reactions and decrease the piezocatalytic performance. A possible reason for this is that at an ultra-high catalyst dose, the collision probability between positive and negative charges is significantly increased by the release of a large number of free charges, decreasing the generation of free radicals and leading to a decline in catalytic performance [48,49,50,51]. The degradation effect of γ-AlON particles on RhB solutions is comparable to the effects of other piezoelectric catalysts [52,53,54,55,56,57].

**Figure 8:**
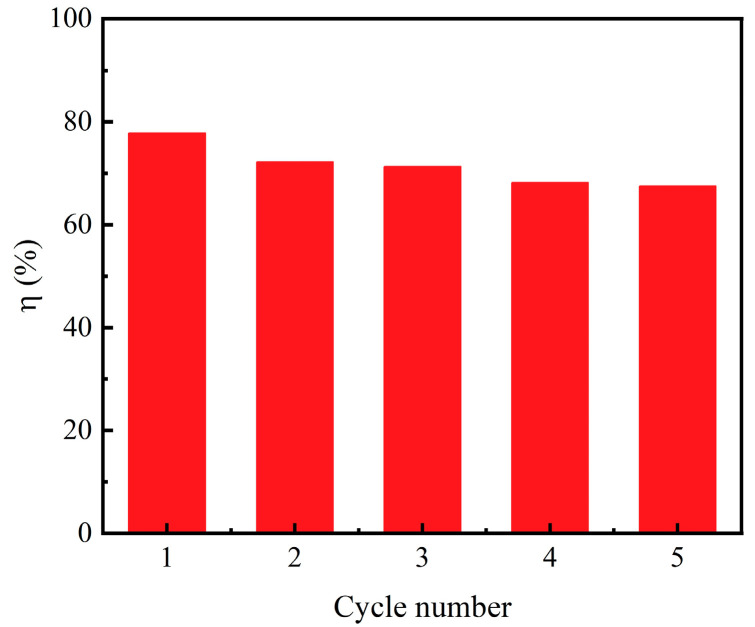
The cycling runs of degradation of RhB by γ-AlON particles.

**Figure 9:**
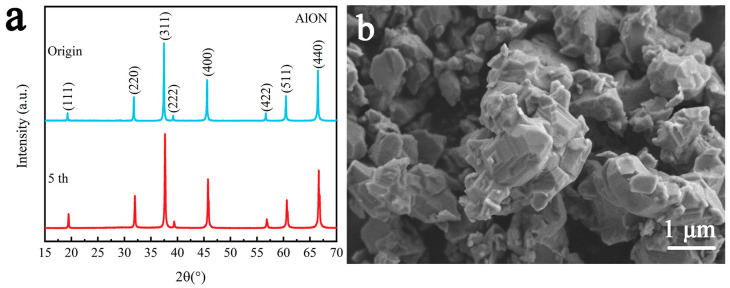
(**a**) XRD pattern and (**b**) SEM image of γ-AlON particles after five cycles of piezocatalytic degradation.

The cycling runs for the degradation of RhB by γ-AlON particles are shown in Figure 8. The initial degradation rate was 78%. As the number of cycles increased, the degradation rate gradually decreased. After five cycles, the degradation rate dropped to 67.5% with a just 13.5% decrease compared to the initial degradation rate. Thus, the γ-AlON particles exhibited a stable durability. As shown in Figure 9a, the XRD pattern of γ-AlON particles after five cycles of piezocatalytic degradation also indicated good crystallinity without new peaks detected. Moreover, it can clearly be seen that there is no change in morphology (Figure 9b). The results imply the high cyclic stability of γ-AlON particles.

**Figure 10:**
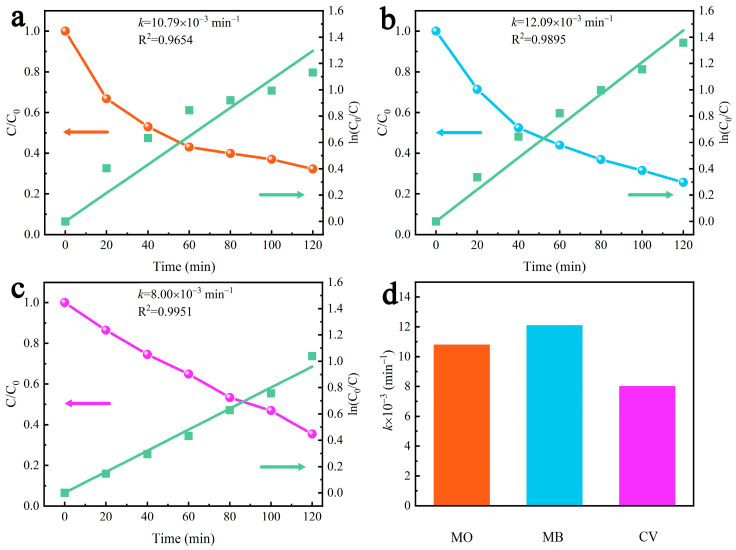
The relative concentration curves and the first-order reaction kinetics curves of (**a**) MO solution, (**b**) MB solution, (**c**) CV solution and (**d**) its rate constant *k* by γ-AlON particles under ultrasonic vibration.

To further evaluate the ability of γ-AlON particles to degrade other dyes, the dose of γ-AlON particles was fixed at 40 mg in 50 mL of dye solution, and MO, MB and CV were employed as target pollutants. The relative concentration (*C*/*C*_0_) of each dye solution decreased with increasing vibration time (Figure 10a–c). After 120 min under ultrasonic vibration, the MO, MB and CV solutions exhibited 67.74%, 74.29% and 64.62% degradation rates, respectively, indicating that the γ-AlON particles exhibited good piezocatalytic degradation ability in various dye solutions. As shown in Figure 10d, the *k* values for the piezocatalytic degradation of the MO, MB and CV solutions were 10.79 × 10^−3^, 12.09 × 10^−3^ and 8.00 × 10^−3^ min^−1^, respectively. The piezocatalytic degradation performance of γ-AlON particles in MB solution was greater than that in the other two dye solutions. Table 1 shows the piezocatalytic performance of various piezocatalysts and compares them with this work. The results show that γ-AlON has good catalytic activity, which indicates that it is a promising novel piezocatalytic material.

### 2.3. Piezocatalytic Mechanism of γ-AlON Particles

The mechanism of the piezocatalytic degradation of dye solutions mainly involves the piezocatalytic generation of active substances such as •OH, •O_2_^−^ and h^+^ in deionised water. These active substances react with large molecular organic compounds to produce water and carbon dioxide, resulting in the degradation of dyes [63].

**Figure 11:**
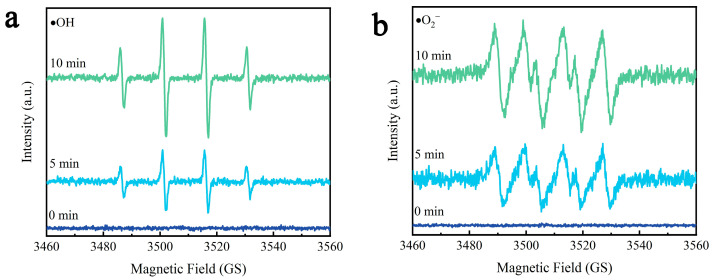
EPR patterns of γ-AlON solutions by DMPO under different ultrasonic vibration times: (**a**) DMPO-●OH signals and (**b**) DMPO-●O_2_^−^ signals.

Piezocatalytic performance can be evaluated through EPR [64], which is an effective technique for detecting the active substances in γ-AlON solutions under ultrasonic vibration. Figure 11 shows the signals for DMPO-•OH and DMPO-•O_2_^−^ in γ-AlON solutions under different ultrasonic vibration conditions. In the initial state without ultrasonic vibration, no significant signals were detected in γ-AlON solutions. The signal intensity increased with the duration of vibration, indicating that γ-AlON solutions generate •OH and •O_2_^−^ under ultrasonic vibration. This result further validates the piezocatalytic performance of γ-AlON particles.

**Figure 12:**
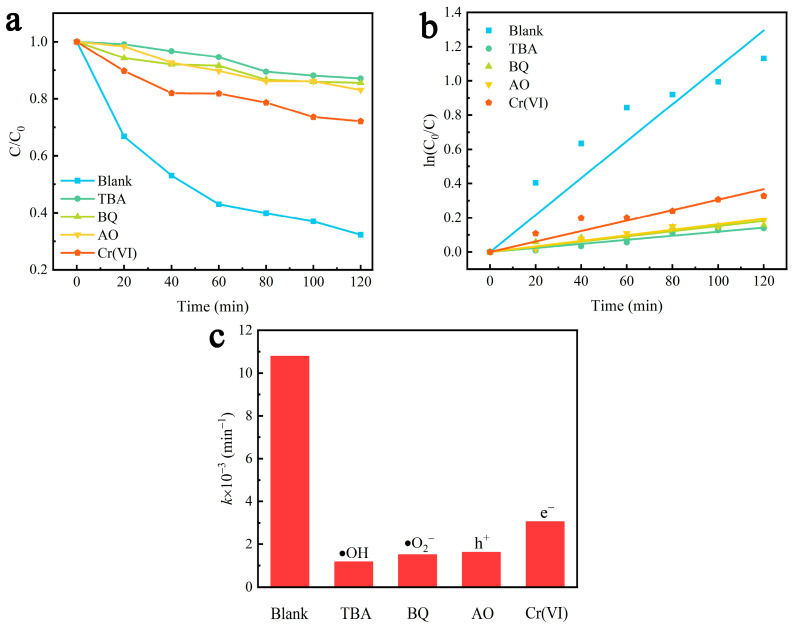
(**a**) The relative concentration curves. (**b**) The first-order reaction kinetics curves and (**c**) the rate constant *k* of MO solutions by γ-AlON particles under ultrasonic vibration with and without free-radical scavengers.

To investigate the effects of active substances on piezocatalytic degradation by γ-AlON particles, MO was selected as the target degradation substance and tert-butanol (TBA, 2 mmol/L), benzoquinone (BQ, 2 mmol/L), ammonium oxalate (AO, 10 mmol/L) and Cr(VI) (potassium dichromate solution, K_2_Cr_2_O_7_, 10 mmo/L) were introduced into the catalytic degradation system as free-radical scavengers of •OH, •O_2_^−^, h^+^ and e^−^, respectively. Compared with the blank sample without free-radical scavengers, samples with TBA, BQ, AO and Cr(VI) exhibited significantly decreased MO degradation efficiency. After 120 min under ultrasonic vibration, the MO solutions with TBA, BQ and AO exhibited 12.93%, 14.44%, 16.91% and 27.92% degradation rates, respectively (Figure 12a). The fitted curves of the degradation kinetics in the free-radical scavenging activity experiments revealed first-order kinetics (Figure 12b). As shown in Figure 12c, the fitted *k* values in the MO solutions with free-radical scavengers significantly decreased. The fitted *k* values for the piezocatalytic degradation of MO solutions in the presence of TBA, BQ and AO were 1.18 × 10^−3^, 1.51 × 10^−3^, 1.62 × 10^−3^ and 3.06 × 10^−3^ min^−1^. However, the fitted *k* value under the same ultrasonic vibration conditions without the free-radical scavengers was 10.79 × 10^−3^ min^−1^. This result indicates that •OH, •O_2_^−^, h^+^ and e^−^ were the main active substances in the piezocatalytic degradation of MO solutions.

**Figure 13:**
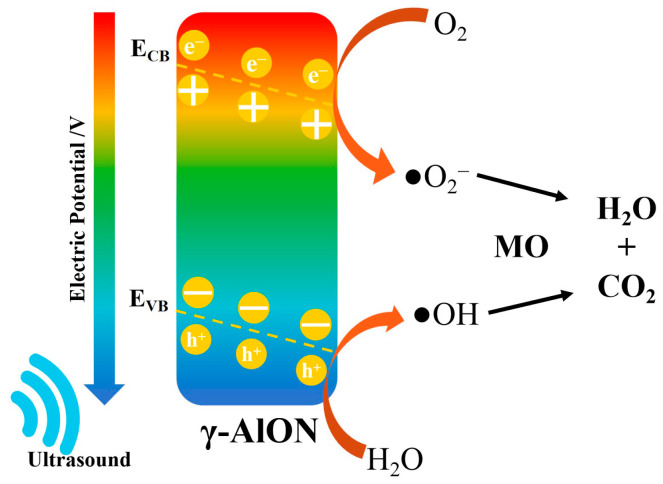
The schematic diagram of piezocatalysis of γ-AlON particles for MO solution.

When AlON particles were employed for the degradation of MO under ultrasonic vibration, the collapse of cavitation bubbles induced by ultrasonic waves could generate pressures as high as 10^8^ Pa [65] at the AlON particle interface, stimulating a positive piezoelectric effect. Meanwhile, the slope of the valence band (E_VB_) and conduction band (E_CB_) is proportional to the piezoelectric potential. Driven by the electric potential, free electrons generated by defects can migrate to the crystal surface [3], resulting in the migration of e^−^ and h^+^ in opposite directions to the AlON surface. The e^−^ and h^+^ generated on the AlON surface will react with O_2_ and H_2_O in water to produce •O_2_^−^ and •OH. MO will be degraded into H_2_O and CO_2_ under the reaction of free radicals of •O_2_^−^ and •OH (Figure 13).

## 3. Material and Methods

### 3.1. Catalyst Preparation

The detailed raw materials for the preparation of γ-AlON particles have been reported in [66]. Firstly, high purity γ-Al_2_O_3_ (94.2 wt%) and activated powder (5.8 wt%) were added to a nylon jar with Si_3_N_4_ balls and absolute ethyl alcohol by planetary mechanical ball-milling at 170 rpm for 24 h. Then, the mixture powder was loaded into a boron nitride crucible and placed in a vertical graphite furnace. Finally, ~5 kg of raw γ-AlON particles could be obtained each time using the CRN method (Equation (1)) at 1550 °C and 1750 °C under the flow nitrogen atmosphere (150 L/h flow rate) for 60 min with ordinary pressure, respectively.

(1) $${Al}_{2}O_{3}\left(s\right)+C\left(s\right)+N_{2}\left(g\right)\to AlON\left(s\right)$$

The synthesised raw γ-AlON particles were subjected to planetary mechanical ball-milling in absolute ethyl alcohol at 250 rpm for 12 h. The resulting slurry was completely dried at 180 °C on a heating plate for 2 h to prepare γ-AlON particles as a novel piezocatalytic material. The analytical grade dyes RhB, MO, MB and CV were purchased from Shanghai Macklin Biochemistry Technology Co., Ltd. (Shanghai, China).

### 3.2. Piezocatalytic Experiments

To investigate the piezocatalysis process, RhB, MO, MB and CV (10 mg/L) were selected as target pollutants. Initially, 20, 40, 60, 80 or 100 mg of γ-AlON particles was added to 50 mL of each dye solution. Before the ultrasonic vibration step, the mixture containing γ-AlON particles and dyes was stirred in the dark for 30 min to allow the dye solutions and γ-AlON particles to reach adsorption equilibrium. Subsequently, the dye solutions were transferred into a jacketed beaker and the beaker was maintained at room temperature using flowing tap water in the ultrasonic vibration source. An ultrasonic vibration source with a power of 500 W and a frequency of 40 kHz from ultrasonic cleaner was employed to apply vibration to γ-AlON particles in the dark. At each 20 min interval, a 4 mL sample of dye solution was extracted and centrifuged to determine its concentration over a total duration of 120 min. The dye concentrations were measured using a visible spectrophotometer at the characteristic wavelength of each dye (RhB-λ_max_ = 554 nm, MO-λ_max_ = 464 nm, MB-λ_max_ = 664 nm and CV-λ_max_ = 582 nm).

To examine the piezocatalytic degradation performance of γ-AlON particles, the concentration and degradation rate (*η*) of dye solutions under different conditions were calculated, the degradation rate constant (*k*) values were determined from the slopes of the graphs by plotting −ln(*C*/*C_0_*) versus ultrasonic vibration time

(2) $$\eta =(1-C/C_{0})\times 100$$

(3) $$ln(C/C_{0})=-kt$$

where *C*_0_ and *C* are the concentrations of dye solutions after dark adsorption and reaction, respectively, and *k* and *t* are the first-order kinetics constant rate and vibration time, respectively.

### 3.3. Characterisation

Structural characterisation of γ-AlON particles was performed using an X-ray analyser (Ultima IV, Rigaku, Tokyo, Japan). A Fourier infrared spectrometer (Nicolet iS20, Thermo, Waltham, MA, USA) was used to analyse the chemical bonds of the γ-AlON particles. The microscopic morphology of the γ-AlON particles was observed using a field emission scanning electron microscope (Ultra 55, Zeiss, Oberkochen, Germany). High-resolution transition electron microscopy (HRTEM) and element mapping of γ-AlON particles were performed using a transmission electron microscope (Talos F200X, Thermo, Waltham, MA, USA). X-ray photoelectron spectroscopy (Scientific K-Alpha, Thermo, Waltham, MA, USA) was used to analyse the chemical composition of the γ-AlON particles. The specific surface area of γ-AlON particles was determined using a surface area and porosity analyser (ASAP 2460, Micromeritics, Norcross, GA, USA). The piezoelectric responses of γ-AlON particles were measured using switching piezoresponse force microscopy (Dimension icon, Bruker, Saarbrücken, Germany). The active substances in the piezocatalytic experiments were identified using an electron paramagnetic resonance (EPR) spectrometer (EMX Plus, Bruker, Germany). The absorbance of the dye solutions at characteristic wavelengths was measured using a visible spectrophotometer (721G, Inesa, Shanghai, China).

## 4. Conclusions

In this study, for the first time, γ-AlON particles prepared using CRN exhibited effective piezocatalytic degradation performance on dissolved dyes under ultrasonic vibration. After 120 min under ultrasonic vibration, 40 mg γ-AlON particles in 50 mL of 10 mg/L RhB, MO, MB and CV achieved 78.06%, 67.74%, 74.29% and 64.62% dye degradation, respectively. The fitted *k* values were 13.35 × 10^−3^, 10.79 × 10^−3^, 12.09 × 10^−3^ and 8.00 × 10^−3^ min^−1^, respectively. The piezocatalytic mechanism of γ-AlON particles was elucidated through free-radical scavenging experiments, which identified •OH, •O_2_^−^, h^+^ and e^−^ as the main active substances. These results illustrate that γ-AlON particles, which can be obtained through cost-effective and high-yield synthesis, are an efficient and promising piezocatalytic material for the treatment of dye pollutants.

## Figures and Tables

**Table 1:** Piezocatalytic performance of various piezocatalysts for dye degradation.

Piezocatalyst	Dye	Initial Concentration (mg/L)	Degradation Ratio (%)	Time (min)	*K* (min^−1^)	Ref.
BT/CuO nanocomposite	RhB MO	5 10	98.4 65.3	120	3 × 10^−2^ 8 × 10^−3^	[58]
15%Ag-ZnO	MO	10	90.5	120	19.58 × 10^−3^	[11]
PZT	RhB	5	90.33	180	no result	[59]
BaTiO_3_ nanocubes BaTiO_3_ nanoparticles	RhB	10	~64 ~68	150	7.6 × 10^−3^ 10.3 × 10^−3^	[60]
BaTiO_3_	MB MO	5	54.1 88.9	180	4.451 × 10^−3^ 10.84 × 10^−3^	[61]
Bi_4_Ti_3_O_12_	MB	5	67	150	7.01 × 10^−3^	[62]
γ-AlON	RhB MO MB	10	78.06 67.74 74.29	120	13.35 × 10^−3^ 10.79 × 10^−3^ 12.09 × 10^−3^	This work

## Data Availability

The original contributions to the study are included in the article, further inquiries can be directed to the corresponding authors.
